# Handmade cloning: recent advances, potential and pitfalls

**DOI:** 10.1186/s40104-015-0043-y

**Published:** 2015-10-15

**Authors:** Geetika Verma, JS Arora, RS Sethi, CS Mukhopadhyay, Ramneek Verma

**Affiliations:** School of Animal Biotechnology, Post Graduate Institute of Veterinary Education and Research, Guru Angad Dev Veterinary and Animal Sciences University, Ludhiana, 141004 Punjab India

**Keywords:** Handmade cloning, Micromanipulator, Somatic cell nuclear transfer, Transgenic, Zona free cloning

## Abstract

Handmade cloning (HMC) is the most awaited, simple and micromanipulator-free version of somatic cell nuclear transfer (SCNT). The requirement of expensive micromanipulators and skilled expertise is eliminated in this technique, proving it as a major revolution in the field of embryology. During the past years, many modifications have been incorporated in this technique to boost its efficiency. This alternative approach to micromanipulator based traditional cloning (TC) works wonder in generating comparable or even higher birth rates in addition to declining costs drastically and enabling cryopreservation. This technique is not only applicable to intraspecies nuclear transfer but also to interspecies nuclear transfer (iSCNT) thus permitting conservation of endangered species. It also offers unique possibilities for automation of SCNT which aims at production of transgenic animals that can cure certain human diseases by producing therapeutics hence, providing a healthier future for the wellbeing of humans. The present review aims at highlighting certain aspects of HMC including recent advancements in procedure and factors involved in elevating its efficiency besides covering the potentials and pitfalls of this technique.

## Introduction

The birth of Dolly in 1997 [1] by the somatic cell nuclear transfer (SCNT) technique was a major scientific innovation in animal cloning. After this, animal cloning by SCNT gained momentum and animal clones from various species were generated (Table 1). Traditional cloning (TC) necessitate the use of expensive micromanipulators for enucleation of recipient oocytes, followed by insertion of a donor somatic cell or nucleus into enucleated oocyte by fusion [2] or direct injection [3] besides requiring skilled expertise. One of the major necessities in TC was to reduce the costs without compromising with the efficiency. Keeping this in view, some adaptations were made in TC to make it more robust and inexpensive, which ultimately led to the birth of handmade cloning (HMC), a technique also known as zona free cloning or hand guided technique [4]. Based on SCNT, HMC is advanced procedure of enucleation of zona-free mammalian oocytes by hand-bisection using a metal blade [5–7] or by employing density gradient centrifugation [8] or by chemicals [9]. The first known zona-free nuclear transfer approach was performed by Tatham et al. [8] but it failed to generate calves. Later on, Vajta et al. [5] developed the improved HMC technique utilizing sharp blades for bisection of zona-free oocytes under steriomicroscope.

**Table 1:** List of animals successfully cloned by either forms of SCNT i.e. TC and HMC

S.No.	Animal	Reference(s)	
		Traditional cloning (TC)	Handmade cloning (HMC)
1.	Cattle	[78, 79]	[38, 50, 80, 81]
2.	Buffalo	[82]	[83]
3.	Goat	[84–87]	--
4.	Sheep	[1]	[73]
5.	Pig	[88]	[69, 89, 90]
6.	Horse	[91]	[92]
7.	Mice	[3]	--
8.	Cat	[93]	--
9.	Rabbit	[94]	--
10.	Rat	[95]	--
11.	Dog	[96]	--
12.	Ferret	[97]	--
13.	Wolf	[98]	--
14.	Mule	[99]	--
15.	Camel	[100]	--

In addition to intraspecies nuclear transfer, HMC has also been exploited for interspecies somatic nuclear transfer (iSCNT). Due to oocyte availability constraints in case of endangered animals, iSCNT is a viable option offering embryo reconstruction by fusing donor cells from the species which needs to be cloned or protected from extinction with recipient oocytes of different animal species. Although some wild animals have been cloned by this method, yet it is less efficient when compared with intraspecies nuclear transfer [10]. It is important to mention that any animal created using either form of SCNT i.e. TC or HMC is not a true clone of its donor because only nuclear DNA of the clone is same to that of donor but not mitochondrial DNA. So, for a clone to be true to its donor, it must inherit nuclear as well as mitochondrial DNA. The present review highlights all the possible recent adaptations incorporated to improve the efficiency of HMC in bovines, caprines and porcines. All the possible mechanisms of performing HMC, in addition to its potential and limitations have been addressed besides, discussing future prospects of this technique.

## Step by step guide to HMC

Both TC and HMC aim at same objective but vary predominantly in their requirements for instrumentation. Table 2 summarizes the similarities and differences between HMC and TC. HMC can be broadly divided into two parts: first dealing with the preparation of donor somatic cell or nuclei and the second dealing with preparation of enucleated oocytes, which is to be fused with donor nuclei later on.

**Table 2:** Summary of similarities and differences between HMC and TC

S.No.	Features	TC	HMC
1.	Somatic cell nuclear transfer	✓	✓
2.	Synchronization of donor cell	✓	✓
3.	High no. of attempts	✓	✓
4.	Problems associated with nuclear reprogramming and reproducibility of experiments	✓	✓
5.	True clone	**×**	**×**
6.	Micromanipulator-free	**×**	✓
7.	Zona- free	**×**	✓
8.	Problems associated with zona pellucida removal	**×**	✓
9.	Number of cytoplast required	Lower	Higher
10.	Costs	Higher	Lower than TC

### Preparation of donor somatic cell or nuclei

#### Source of donor nuclei

HMC was initially established for bovine nuclear transfer using embryonic cells as source of donor nuclei [11]. However, with continuous experimentation in this field many other sources have been explored. Various sources are listed in Table 3. Although any somatic cell can be use as a donor for HMC, yet choice of donor cells is very essential to overcome the problems related to nuclear reprogramming. Saini et al. [12] has observed that donor cells of fibroblast origin are easier to reprogram than those of epithelial origin in iSCNT through HMC. Primary cell cultures are established aseptically following standard procedures depending upon the source.

**Table 3:** List of sources of donor nuclei for HMC

S.No.	Source of donor nuclei	Species	Reference(s)
1.	Pronuclear stage embryos	Mouse	[101, 102]
2.	Embryonic blastomeres	Mouse	[65]
3.	Cumulus cells	Cow	[4, 5]
4.	Embryonic stem cells (ESC)	Buffalo	[60]
5.	Adult fibroblast cells	Buffalo	[52]
		Cow	[47, 58, 103]
		Goat	[10, 12, 54]
		Sheep	[73]
6.	Natural killer T cells	Mice	[104]
7.	Fetal fibroblasts	Goat	[55, 85, 105]
		Water buffalo	[106]
		Pig	[69]
8.	Adipose tissue derived-Mesenchymal stem cells	Goat	[107]

#### Synchronization between donor nucleus and the recipient cytoplasm

The synchrony between the cell cycle of donor nucleus and the recipient cytoplasm is considered to be one of the key factors needed to increase nuclear reprogramming capacity and, thereby, cloning efficiency [13]. The use of quiescent donor cells in the G0 or arrested in the G1 phases of the cell cycle has become a rule in cloning [14], since such cell cycle phases are considered as more suitable for proper reprogramming [13, 15–18]. In addition, the use of donor cells at other cell cycle phases usually leads to poor embryo development after cloning due to either chromosome pulverization induced by premature chromosome condensation when occurring in S phase, or aneuploidy in G2/M phases [13].

The different procedures for the cell cycle synchronization of donor cells in G0/G1 phase includes use of serum starvation, contact inhibition by cell confluence, and use of chemicals like cycloheximide, DMSO, and roscovitine. Among all the different procedures, cell confluence by contact inhibition appears to be one of the most widely used methods nowadays [19, 20] as the proportion of cells in G0/G1 appeared to be higher by cell confluence than any other protocol. Moreover, serum starvation or the use of chemical agents for cell cycle synchronization prior to somatic cell cloning not only impose a potential stressful condition on cells, but also do not appear to promote any improvement in embryo development [21].

### Preparation of recipient enucleated oocytes/cytoplasts

#### Source of oocytes

Oocytes can be retrieved either from live animal (*in vivo*) or slaughtered animal (abattoir). Oocytes have been obtained *in vivo* from hormonally stimulated females by laparoscopic oocyte recovery (LOR) or transvaginal oocyte retrieval (TVOR), also known as Ovum pick up (OPU) or from slaughterhouse ovaries from nonstimulated females by post-mortem [22]. Although oocytes in large numbers are available in case of slaughtered animal yet it suffers from a limitation of absence of information about pedigree of dam which is available in case of OPU.

#### *In vitro* maturation (IVM) of oocytes

Mammalian oocytes are arrested at the diplotene stage of the first meiotic division. In response to the preovulatory surge of gonadotrophins some of the oocytes undergo resumption of meiosis characterized by germinal vesicle breakdown, chromosome condensation, formation of the first meiotic spindle, expulsion of the first polar body and arrest in metaphase of the second meiotic division. These events are defined as oocyte maturation and lead to an ovulated fertilizable oocyte. For IVM, oocytes are incubated for upto 24 h (bovines) [23, 24]; 27 h (caprines) [22, 25] and 44 h (porcines) [26] in medium supplemented with hormones (gonadotropins) and growth factors under mineral oil at 39 °C in 5 % CO_2_ in humidified air. Gonadotrophins play a major role in triggering resumption of meiosis in the oocyte and expansion of cumulus oophorus.

#### Zona pellucida removal

After IVM, matured oocytes are selected on basis of cumulus expansion or Giemsa staining. Cumulus-oocyte complexes (COCs) are denuded by pipetting or vortexing [27]. Denuded oocytes are subjected to 0.5 % protease for zona pellucida removal. Zona pellucida can also be removed by pronase treatment [28] or hyaluronidase treatment [4].

#### Enucleation of zona- free oocyte and Cytoplast selection

Zona-free oocytes can be enucleated by chemicals, density gradient centrifugation or by hand using a sharp blade. A non-invasive chemical enucleation procedure was first described by Fulka and Moor [9] for mouse oocytes in which chemical treatment blocks DNA topoisomerase II during metaphase-I thus, inhibiting the oocyte chromosomes separation and expelling the whole chromatin into the first polar body, resulting in a chromatin-free cytoplast, also known as chemically enucleated oocyte (CEO) [29].

A brief treatment of bovine [30]; porcine [31] and caprine [32] oocytes with demecolcine (DEM) produces a membrane protrusion that contains a mass of condensed chromosomes, which can be removed easily. Other chemicals such as nocodazole, etoposide, caffeine, and MG132, have been used to induce or assist oocyte enucleation [31]. Tatham et al. [8] utilized density-gradient centrifugation for enucleation of zona-free oocytes. Vajta et al. [5] developed superior HMC technique employing sharp blades for bisection of zona-free oocytes by hand, hence the name HMC. After removal of zona pellucida, a cone protrudes out of the zona-free oocyte referred as protrusion cone or oocyte rod. Protrusion cone oriented bisection is done using an embryo-splitting blade under a stereomicroscope to obtain zona-free cytoplasts. The protrusion cone or oocyte rods should be bisected near the end of the rod as it is the place where the polar body is located [29]. If the polar body is lost or unable to locate or the egg is a zygote it is better to dissect the egg into two equal halves to generate cytoplasts [33]. After bisection, all halved-oocytes without chromatin, known as cytoplasts or hemi-cytoplasts, are selected by screening for nuclear material stained with Hoechst 33342 under ultraviolet (UV) light. However, use of UV may prove detrimental to oocytes. Moreover, lipid droplets in the ooplasm obstruct identification and enucleation of metaphase II (MII) chromosomes, especially in bovine species, ultimately affecting the efficiency of the process. To overcome this problem, a new experimental system have been employed, which is based on fluorescent observation of chromosomes in living oocytes without any damage by injecting fluorescence-labeled antibody conjugates that bind to chromosomes and fluorescent observation using a conventional halogen-lamp microscope [34].

#### Embryo reconstruction and activation

**Fig. 1:**
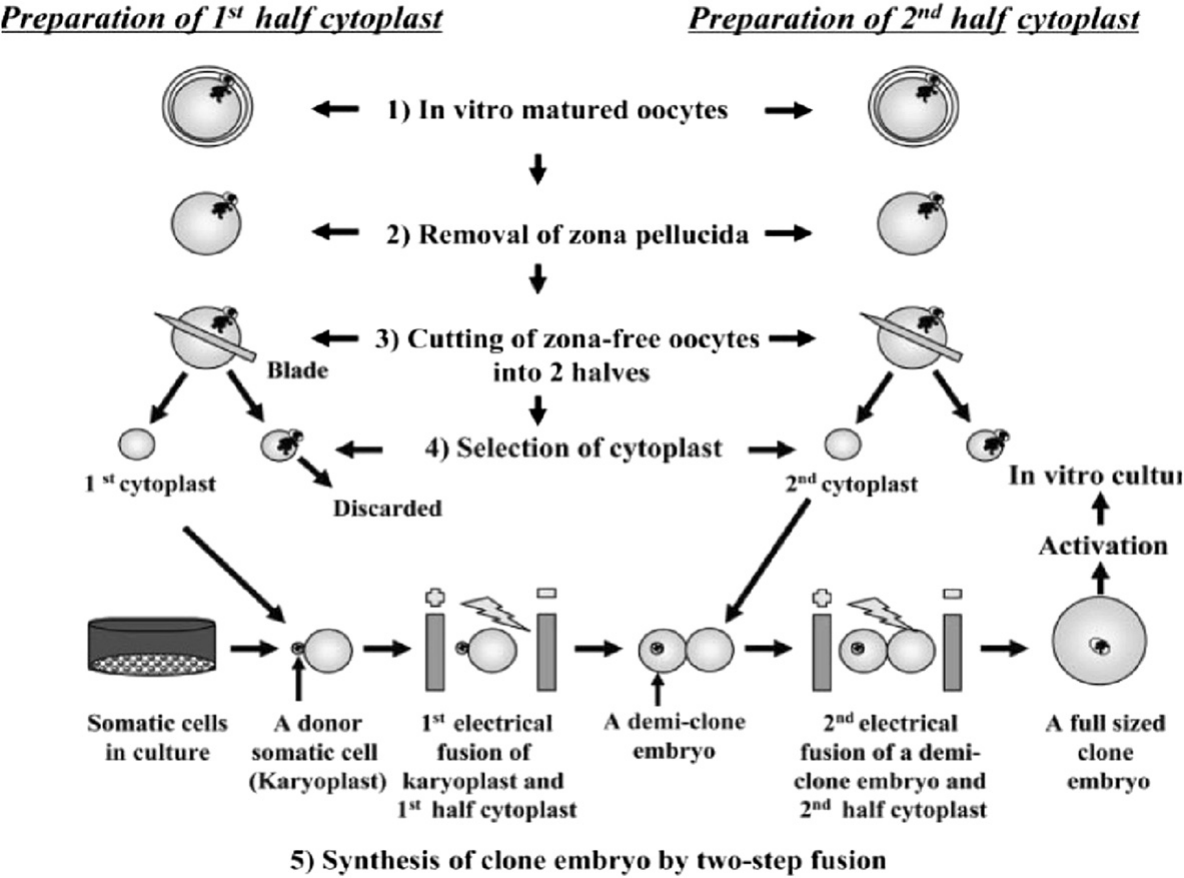
Outline showing procedures for Handmade cloning (HMC) (Reprinted from Nagai et al. [33]). Figure shows (1) the preparation of cytoplast by removal of cumulus layer of in-vitro matured oocytes (2) removal of zona pellucida from oocyte by digestion with pronase (3) hand bisection of zona-free oocytes with a blade, hence the name hand-made cloning, to obtain cytoplast (4) selecting a cytoplast under stereomicroscope (5) reconstructing embryo by fusing a donor somatic cell with cytoplast generating a demi-clone embryo which is again fused with 2nd half cytoplast (generated by same procedure as 1st cytoplast) to obtain a full sized clone embryo that is activated and implanted at blastocyst stage into recipient animal

Reconstruction and nuclear transfer is achieved by quickly exposing one or two enucleated hemi-cytoplasts to phytohemoagglutinin (PHA). PHA helps in attachment of donor cell with cytoplasts by gluing the surface of cytoplast and makes it sticky to easily get attached to the surface of donor cell, leading to formation of a couplet. The couplet may achieve approximately either 50 or 100 % of the final cytoplasmic volume, depending upon one cytoplast or two cytoplasts fusion respectively. After attachment, reconstructed hemi-embryos (50 % volume) or embryos (100 % volume) are fused either by single step electrofusion or two step electrofusion. A single low-voltage AC pulse in electrofusion medium is given in a chamber coupled to an electrofusion apparatus. Vajta et al. [28] make use of two electric fusions as shown in Fig. 1. The cytoplast is dropped over a single somatic cell or karyoplast and exposed to electric pulse. The pair (demi-clone embryo) is removed and then subjected to second electrical fusion involving a demi-clone embryo and second half cytoplast. This technique requires more number of oocytes as a source of recipient cytoplasts than a single step electrofusion. Electrofusion is followed by chemical activation of reconstructed clone embryos in ionomycin and N-6 dimethylaminopurine (6-DMAP) [21]. Ionomycin induces calcium release which suppresses maturation promoting factor (MPF) and 6-DMAP is a protein kinase inhibitor that prevents reformation of MPF, hence resulting in release from metaphase.

#### *In vitro* culture (IVC)

Cloned embryos are then cultured *in vitro* individually in microwells using the well-of-the-well (WOW) system. WOW culture system provides three dimensional blastomere arrangements in zona-free embryos [35]. Activated cloned embryos (1 × 100 %) or two cloned hemi-embryos (2 × 150 %, embryo aggregation) are placed in each microwell (WOW). All structures are cultured *in vitro* to the blastocyst stage. Cleavage rate is assessed 48 h after activation [21]. It has been observed by Kaith et al. [36] that embryos which cleave early have higher developmental competence and quality than those that cleave relatively later.

#### Assessment of embryo quality, stage of development and cell density

Embryo quality and stage of development are assessed by morphology according to the guidelines from the International Embryo Transfer Society (IETS). In the case of zona-free embryos, especially for expanded and hatched blastocysts, stages of development are assessed by comparing embryo size and morphology with blastocysts from the zona-intact control groups. This is done by exposing blastocysts to Hoechst 33342 under UV light in an epifluorescent microscope.

#### Embryo transfer

After assessment of embryo’s quality and stage, it is subjected to transfer into recipient animal. Embryos are transferred on day seven for cattle, goat, pig and day eight for buffalo to synchronised recipient donors.

#### Embryo vitrification and in-straw thawing

Based on assessment of embryo quality, stage of development and cell density, the embryos of good quality can be cryopreserved for long time in liquid nitrogen. Blastocysts are vitrified using the open-pulled straw (OPS) method as described by Vajta et al. [37], which resulted in healthy offspring after transfer [38]. Considering the delicacy of zona-free embryos, modification of the vitrification procedure may be required to achieve the commercially viable level of efficiency [27]. Embryos are thawed using the in-straw dilution method prior to embryo transfer [39, 40].

## Factors contributing to high efficiency of HMC *in vitro*

### Effect of method adopted for enucleation of oocyte

Chemically assisted enucleation has been observed as more efficient and reliable method for goat HMC as compared to oriented bisection of oocyte by hand [41]. DEM treatment disrupts spindle microtubules, induces chromosome condensation and has significantly greater potential than that of embryos generated from mechanically enucleated oocytes *in vivo* [31]*.* The rates of enucleation, cell fusion and blastula formation of goat oocytes has been found to be similar among caffeine- and DEM-assisted enucleation and significantly higher than mechanical enucleation as reported by Wang et al. [42]. DEM-assisted enucleation is advantageous over mechanical enucleation as it decreases the cytoplasmic volume of the oocyte minimally without reducing the level of MPF. High MPF activity might promote cytoplasmic maturation and improve the developmental competence of oocytes [31]. Moreover, enucleation with chemicals like DEM obviates the need of inverted fluorescence microscope and destructive chromatin staining and UV irradiation for cytoplast selection. However, contrary to it, oriented bisection by hand has been found more promising than chemical assisted enucleation in case of fibroblast derived transgenic porcine embryos [43]. Similarly, the production of bovine blastocysts using oocytes enucleated by DEM showed lower embryonic developmental rates than those obtained with mechanically enucleated oocytes [44]. DEM treatment has negative effect on the completion of second polar body extrusion, which results in a significant number of partially enucleated oocytes in which the chromosomes eventually reintegrate into the cytoplasm [44, 45]. However, the shorter treatments with DEM or the use of alternative microtubule disrupting drugs may solve these problems. These alternative drugs must be chosen carefully to minimize their detrimental effects on the developmental potential of the cytoplasts [46].

### Effect of final cytoplasmic volume of fused hemicytoplasts

The effect of the fusion of hemicytoplasts or aggregation of hemiembryos, varying the final cytoplasmic volume, on development and cell density of embryos produced by HMC has been evaluated by Ribeiro et al. [47]. The increase in cytoplasmic volume, either by fusion or by aggregation, had a positive effect on embryo development, supporting the establishment of pregnancies and the birth of a viable clone calf after transfer to recipients. It has been shown that the increase in the number of aggregated structures within each WOW follows a linear increase in cleavage, blastocyst rate, and cell density. The quality of handmade cloned porcine embryos has been improved by multiple embryo aggregations [48]. However, hemi-cytoplasts with cytoplasmic volume (∼85 % *vs.* 2 × 50 %) showed no effect on fusion rates after embryo reconstruction, rendering it advantageous for species such as the goat, for which oocyte supply and numbers are usually limiting factors [22].

### Effect of zona pellucida removal

As only small, round and intact cells, with visibly soft membranes, are used for embryo reconstruction [49] so zona pellucida removal is an important parameter and it has been found that pronase digestion in the presence of serum is an efficient and harmless method for removal of zona pellucida, even when large quantities of oocytes (up to 150) are digested together [4]. Moreover, fluidity of zona-free eggs should be maintained in order to prevent egg lyses. Elsheikh [29] observed that on culturing zona-free eggs in media supplemented with Cytochalasin B, their cytoplasm generally becomes more fluid and thus deforming them into cylindrical rods. In addition, suction of eggs into the deforming pipette should be done carefully, slowly and very smoothly to avoid egg lyses. The end of this pipette should be well fire-polished to avoid injury of the zona-free eggs. However, Oback et al. [50] found no significant difference in development to live calves between zona-free and zona-intact embryos derived from the same adult fibroblast line. Blastocysts of transferable quality were obtained at similar rates from zona-free and control zona-intact embryos.

### Effect of cytoplast and donor somatic cell fusion

It has been observed by Vajta et al. [4] that in electrofusion, the cytoplast-somatic cell fusion pairs should be placed between the wires of the fusion chamber for significantly higher rates of fusion than in a random position or with somatic cells furthest from the wire. Also, the second fusion is successful regardless of the time between the first and second fusion. But, if the second fusion pulse occurs before completion of the first fusion, the first fusion could be disturbed, even if the second fusion has completed successfully. Elsheikh [29] observed that during aggregation of cytoplasts and donor nuclei the area of contact between them should be wide to achieve high fusion rates.

### Effect of different culture media and culture systems

The cloned embryo culture medium has always been very important. In the zona-free method the frequent change of medium during culture, is not preferred [51]. Cleavage and blastocyst rates for zona-free cloned buffalo are higher in Research Vitro Cleave (RVCL; Cook®, Australia) medium compared to modified Charles Rosenkrans 2 (mCR2) and modified Synthetic Oviductal Fluid (mSOF) medium as observed by Shah et al. [52]. Selokar et al. [53] performed interspecies handmade cloned embryo production, by nuclear transfer of cattle, goat and rat fibroblasts to buffalo oocytes, using RVCL as the culture medium. Dutta et al. [54] used RVCL media to produce zona-free cloned goat embryos. North Carolina State University (NCSU) medium has been used for porcine embryos [26, 31]. Studies on zona-free cloned buffalo [52] and goat embryos [55] revealed that flat surfaces (FS) used as culture system yielded significantly higher blastocyst rates than WOW or microdrops (MD). Also, development in WOW has found to be significantly better than MD. Vajta et al. [4] studied the effect of two common constituents of the bisection medium used for embryo manipulation: sucrose, an osmotic buffer, and cytochalasin B, a cytoskeleton relaxant. Sucrose incubation has found to decrease the percentage of demioocytes surviving bisection with an intact cell membrane whereas Cytochalasin B increases the survival.

### Other factors enhancing the blastocyst/oocyte rates

Blastocyst/oocyte rates can be enhanced by prolonged incubation between reconstruction, application of serum-containing medium for Ca-ionophore incubation, and individual incubation of reconstructed zona-free embryos in DMAP. These changes decrease lysis rates and prevent unintended attachment of embryos to each other [4]. Zona-free embryos are more sensitive to chemical activation [6] so, a low concentration (2 mmol/L) of Ca-ionophore can be employed. The incubation period between reconstruction and activation and during DMAP exposition should be prolonged from 3 to 4 h and from 4 to 6 h, respectively, according to the observations of Alberio et al. [56], Kasinathan et al. [57] and Beyhan et al. [58].

Supplementation of calf serum (CS) in culture medium for *in vitro* fertilised embryos has been found to significantly increase the blastocyst/oocyte rate as compared to bovine serum albumin (BSA) as observed by Vajta et al. [4]. Reprogramming of donor cells by pre-treating them with oocyte extracts and selection of developmentally competent oocytes through brilliant cresyl blue (BCB) staining for recipient cytoplast preparations may enhance expression of developmentally important genes in hand-made cloned embryos at levels similar to *in vitro* fertilized counterparts [59]. Similar findings have been demonstrated by Mohapatra et al. [60], Rodríguez-González et al. [61] and Roca et al. [62] for selecting buffalo, prepubertal goat and pig oocytes respectively using BCB staining.

## Problems limiting the success of HMC

The problems that limit the success of HMC are mainly associated with automation of the technique and with use of zona-free embryos. These are discussed in detail here.

### Problem associated with automation of the technique

The automation of HMC procedure can make cloning easier, simpler and time-saving technique and it can be achieved by use of microchannels. Efforts have been made by Vajta et al. [63] using microchannels. However, there is still lack of integration of the individual steps into a production line and unfortunately, attempts in this field are meagre which present a main hurdle in the advancement of this field [64]. Efforts are needed to overcome the existing fundamental drawbacks like the occurrence of gas bubbles in the channels during incubation, hampering the passage of solutions and deformation of the embryos. Hence, an advanced technical setting to control, fine-tune and integrate processes is required.

### Problems of zona-free embryos

Normal embryos are equipped with zona-pellucida which protects them not only from toxic substances in culture media [65] but it is essential for even undisturbed embryonic development [65, 66] besides preventing separation of blastomeres in cleaving embryos. Difficulty in obtaining newborns from zona-free embryos has been reported [67]. The rates at which zona-free blastomeres separated at the 2-cell stage developed to the blastocyst stage and to full term were lower than those of zona-intact 2-cell embryos [68]. Further, the cell number of inner cell mass (ICM) in blastocysts derived from zona-free 4-cell embryos has been found lower resulting in low rates of implantation [66] which can be overcome by using WOW technique that provides three dimensional blastomere arrangement in zona-free embryos [35]. The zona-free embryos might be affected by toxic substances in culture media. Furthermore, their development might be disturbed. These problems can be overcome by using an artificial zona pellucida [65].

## Benefits of HMC

### High efficiency and productivity

HMC is highly efficient or even superior to TC. Studies to produce transgenic pigs that express the functional nematode fat-1 gene have been conducted by Zhang et al. [69]. After 7 days of culture *in vitro*, 37 % of reconstructed embryos developed to the blastocyst stage by HMC and a total of 14 live offspring were produced, which is more efficient than TC. Using HMC, about 100–140 reconstructed embryos can be generated by just two qualified technicians in 2.5–3 h [70]. Having this large number of embryos correlates to higher probability of efficient transfer and high productivity.

### Reduced labour and costs

In contrast to the complicated tools, like micromanipulators and microscopes which are required for TC, only one stereomicroscope and one electrofusion machine are required for HMC, making it simple and cost-effective technique. For reconstruction of embryos, two step fusion can be reduced to one step which reduces requirement of more oocytes and hence, reduces labour [21].

### Time saving

Fortunately, HMC embryos can be cryopreserved successfully with vitrification. Initial reports suggest no decline in pregnancy rates after cryopreservation [64]. Pregnancy rates of around 50 % can be achieved with cloned zona-free embryos in cattle [71]. As per the existing data, no significant difference in the rate of developmental anomalies between TC and HMC was observed in cattle. Moreover, in TC single oocyte can be processed at a time while in HMC multiple oocytes can be processed, hence saving time.

## Future perspectives

HMC holds a promising future. In the future improved HMC programs could be developed for the production of cloned animals with desired traits, rescue of endangered animal species and create transgenic animals for medical purposes like xenotransplantation.

### Conservation of endangered species

HMC allows both interspecies and intraspecies nuclear transfer. Being more efficient and time saving than TC, efforts can be made in exploiting HMC to generate large number of interspecies embryos for conserving endangered species. Studies has shown that not only, the interspecies embryos of goat can be produced using sheep oocytes as donor cytoplast but also the percentages can be improved by using RVCL media for culturing of the embryos as illustrated by Khan et al. [10]. Wild buffalo embryos by iSCNT through HMC using somatic cells of wild buffalo and oocytes of domestic buffalo have been generated [11, 72]. However, more studies in this area are required to optimize media, culture conditions and validation of embryo development for iSCNT by HMC.

### Production of transgenic animals

Transgenic animals, expressing a gene of interest, can be generated by this technique which will surely boost up the field of therapeutics and medicine, besides, contributing organs for xenotransplantation. HMC has been exploited to produce transgenic sheep with elevated levels of omega-3 fatty acids [73] and transgenic pigs expressing the functional nematode fat-1 gene [69]. However, using HMC, little work has been done in this context. For therapeutic purposes, patient-specific embryonic stem cells are required i.e. the cells must be genetically identical to patient, which is a problem of reconstructed embryos, that need to be addressed in this field.

### Automation of HMC

The utmost benefit of HMC is large scale production. Microchannel or microfluidics technology can help in this. Almost all the steps required for HMC can be performed in microchannels [74]. Contrary to it, automation seems to be impossible in TC. This would lead to a revolution for SCNT and all embryo technologies, ensuring production of high grade embryos.

### Nuclear reprogramming studies

Although HMC and TC have been used for cloning many animal species yet both are surrounded with certain limitations including cloning syndrome (developmental abnormalities), poor embryo survival rate, lack of understanding of the mechanism(s) of reprogramming of the transferred somatic cell nucleus [33], placental abnormalities, pregnancy losses and reproducibility of the experiments [75–77]. Detailed studies of underlying mechanisms of reprogramming need to be done in order to overcome above hindrances.

## Conclusions

HMC has emerged as simplified alternative to traditional cloning. It is as efficient as or even higher than micromanipulator-based SCNT in animals. This technique is devoid of micromanipulators and has shown promising prospects of low cost production of genetically modified animals. A little more efforts in this field are required to dissipate this technology in every sector of livestock for all species.

## Abbreviations

SCNT: Somatic cell nuclear transfer
HMC: Handmade cloning
TC: Traditional cloning
iSCNT: Interspecies nuclear transfer
FS: Flat surface
WOW: Well of the Wells
MD: Microdrops
RVCL: Research Vitro Cleave
mCR2: Modified Charles Rosenkrans 2
mSOF: Modified Synthetic Oviductal Fluid
LOR: Laparoscopic oocyte recovery
TVOR: Transvaginal oocyte retrieval
OPU: Ovum pick up
CS: Calf serum
BSA: Bovine serum albumin
BCB: Brilliant cresyl blue
ICM: Inner cell mass
6-DMAP: N-6 dimethylaminopurine
IETS: International Embryo Transfer Society
ESC: Embryonic stem cells
COCs: Cumulus-oocyte complexes
OPS: Open-pulled straw
PHA: Phytohemoagglutinin
CEO: Chemically enucleated oocyte
UV: Ultraviolet
MPF: Maturation promoting factor
IVM: *In vitro* maturation
MII: Metaphase II
IVC: *In vitro* culture
DEM: Demecolcine
NCSU: North Carolina State University
